# Neural effects of a single dose of fluoxetine on resting-state functional connectivity in adolescent depression

**DOI:** 10.1177/0269881120959608

**Published:** 2020-11-04

**Authors:** Liliana P Capitão, Robert Chapman, Nicola Filippini, Lucy Wright, Susannah E Murphy, Anthony James, Phil J Cowen, Catherine J Harmer

**Affiliations:** 1 Department of Psychiatry, University of Oxford, Oxford, UK; 2 Oxford Health NHS Foundation Trust, Oxford, UK; 3 Oxford Centre for Human Brain Activity, Wellcome Centre for Integrative Neuroimaging, Department of Psychiatry, University of Oxford, Oxford, UK

**Keywords:** Fluoxetine, adolescent depression, resting-state functional connectivity

## Abstract

Fluoxetine is commonly prescribed in adolescent depression, but the neural mechanisms underlying its action remain poorly understood. Here, we used resting-state functional magnetic resonance imaging to investigate the effects of a single dose of fluoxetine vs. placebo in adolescents with major depressive disorder. In contrast with previous studies in adults that have demonstrated an acute effect of antidepressants on activity within the default mode network, a single dose of fluoxetine did not alter activity in this network in adolescent depression. There were unexpected group activity differences in the motor network, which should be clarified in future research.

## Introduction

Fluoxetine, a selective serotonin reuptake inhibitor (SSRI), is the first-line drug treatment for adolescent depression, but the neural mechanisms underlying serotonergic antidepressants in this age group are still poorly understood. The use of resting-state functional magnetic resonance imaging (rsfMRI) has proved useful in characterising the effects of SSRIs on neural networks in both healthy volunteers and clinical populations. This approach has the advantage of removing the effects of confounding variables associated with task-based studies such as differences in understanding task instructions and in behavioural performance. It also allows an easier comparison between different studies, as resting-state protocols tend to be more uniform and easier to replicate.

In adults, long-term treatment with SSRIs has been found to normalise activity within the default mode network (DMN) (Posner et al., 2013). In line with the adult literature, a recent study by Cullen and colleagues (2016) found that clinical improvement following SSRI treatment in adolescents was associated with a reduction in connectivity between the amygdala and the precuneus/posterior cingulate, part of the DMN network, an effect that may help attenuate the tendency of adolescent patients to engage in negative thinking. Treatment improvement in this study was also associated with an increase in the connectivity between the amygdala and frontal regions. However, these results are still preliminary and were found at a time when concurrent mood changes were already apparent. The current study therefore aimed to assess the effects of fluoxetine on functional connectivity in adolescents with depression using rsfMRI after a single acute dose vs. placebo, prior to change in symptoms. Based on previous adult and adolescent studies, we predicted that a single dose of fluoxetine would alter activity in the DMN.

## Methods

### Participants

Thirty-one adolescents with a primary

diagnosis of major depressive disorder (MDD), as measured using the Schedule for Affective Disorders and Schizophrenia for School-Age Children **–** Present and Lifetime version (Kaufman et al., 1997), were recruited from Child and Adolescent Mental Health Services (CAMHS). CAMHS psychiatrists made the clinical decision to initiate fluoxetine treatment. Participants were excluded if they presented with a history of bipolar disorder or schizophrenia, substance abuse, current use of psychotropic/antidepressant medication, pregnancy and/or contraindications to MRI.

A formal sample size calculation was precluded, because no prior study had determined the acute effect of fluoxetine on brain activity in depressed adolescents. Our previous work showed that a single dose of fluoxetine reduced facial recognition of anger, with an effect size of 0.81 (Capitão et al., 2019). In a previous functional MRI (fMRI) study in healthy adult volunteers, a single dose of the SSRI citalopram was found to reduce amygdala activation with an effect size of 1.19 (Murphy et al., 2009). Informed by these data, an a priori sample size calculation for the current between-subjects design yielded *n* = 13 per group as the minimum sample size required to detect neural activity differences (difference between two independent means: two tailed, alpha = 0.05, effect size = 1.19, power = 0.8).

### Procedures and measures

For a detailed description of the procedures and measures used in this study, please refer to Capitão et al. (2019). Eligible patients were randomised to receive a single dose of either liquid fluoxetine (10 mg) or a matched placebo (peppermint syrup) in a double-blind procedure. The scan started 6 h after dosing, at a time where the plasma concentration of fluoxetine was expected to be at its peak. Participants were asked to complete a faces task and an emotional regulation paradigm (described elsewhere) and then a resting-state paradigm. During the 10-min resting-state scan, participants were instructed to look at a fixation cross, think of nothing in particular and not fall asleep.

### Resting-state functional connectivity

#### Independent component analysis

Independent component analysis (ICA) was carried out using Multivariate Exploratory Linear Optimized Decomposition into Independent Components (MELODIC, part of FSL: http://www.fmrib.ox.ac.uk/fslmelodic/). For a more detailed characterisation of the preprocessing steps see Filippini et al. (2009).

At an individual level, all components were manually classified as either signal or noise. Components defined as noise were subsequently removed. The preprocessed cleaned functional data were then temporally concatenated across subjects in order to create a single 4-dimensional dataset. The group-wise concatenated multiple rsfMRI datasets were decomposed using a group ICA to identify large-scale patterns of functional connectivity in the population of subjects (restrained to 25 components) as described previously (Filippini et al., 2009). Components corresponding to known resting-state networks (RSNs) were then identified.

The subject-specific analysis of the resting-state data was then carried out using dual regression (Filippini et al., 2009), a technique that allows for voxel-wise comparisons of resting-state functional connectivity between subjects or subject groups. The resulting spatial maps were then tested using a voxel-wise general linear model-based analysis to assess group differences (placebo vs. fluoxetine) using permutation-based nonparametric testing (5000 permutations). Clusters were determined by using threshold-free cluster enhancement and a family-wise error-corrected cluster significance threshold of *p* < 0.05.

#### Seed-based functional connectivity analysis

The denoised functional data from the ICA analysis was used in the seed analysis. Masks of the left and right amygdala were created from the probabilistic maps provided by the Harvard-Oxford Subcortical Structural Atlas within FSL. Masks of an area of white matter (WM) and cerebrospinal fluid (CSF) were also created. Inverse registration maps were used to convert all masks from standard space into each individual’s functional space. The mean time series for voxels within the masks was calculated for each individual.

Seed-based correlations were carried out for each participant using FMRI Expert Analysis Tool (FEAT) v6.0. The time series derived from the mask corresponding to the relevant seed (i.e. left amygdala or right amygdala) was included as an explanatory variable to determine which other brain areas significantly correlated with this time course. Time series derived from WM and CSF masks were included as confound regressors.

Group FEAT analyses were then run for each seed, to determine whether fluoxetine administration affected functional connectivity with the seed. This analysis utilised FMRIB’s Local Analysis of Mixed Effects. Statistical maps were thresholded using clusters determined by *Z* > 3.1 and a corrected cluster significance threshold of *p* < 0.05.

## Results

### Demographic and clinical characteristics

For a detailed characterisation of the demographic and baseline clinical characteristics of this sample, please refer to Capitão et al. (2019). The final analysis consisted of 29 participants, as 2 participants did not complete the scan successfully. The groups were well matched in the baseline clinical measures such as age, gender distribution, IQ, family income, mean age of depression onset, number of comorbidities, depression severity, trait anxiety and suicidal ideation.

### Resting-state functional connectivity

**Figure 1.**
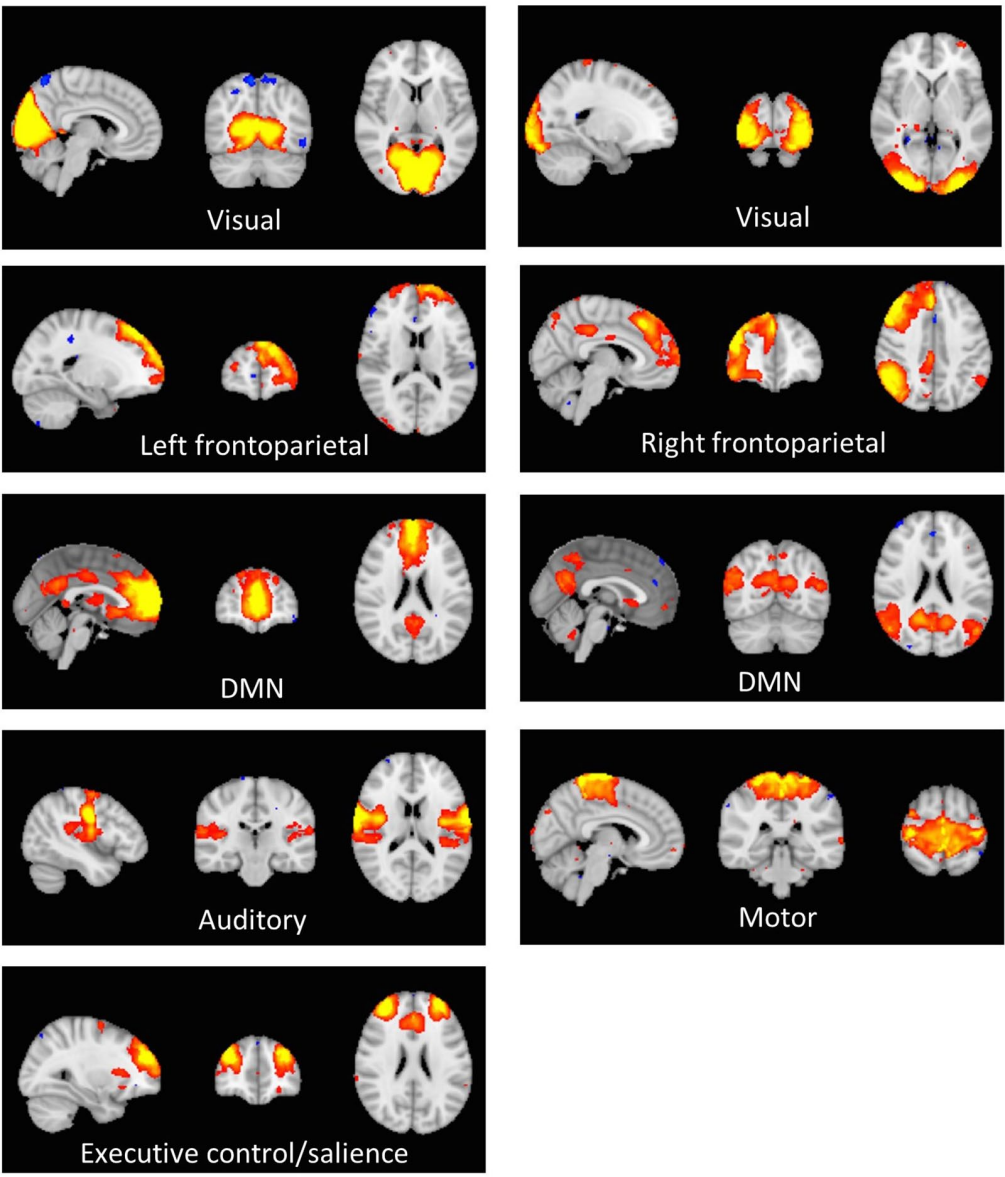
Axial, coronal and sagittal slices of the main RSNs detected, overlaid onto the standard Montreal Neurological Institute brain.

Of the 25 prespecified components, 18 components were identified as RSNs and were evaluated further (Figure 1). The other components reflected distinct artefacts resulting from head motion and physiological or scanner noise. The RSNs of interest included the anterior and posterior DMN, medial and lateral visual, auditory, motor, executive control/salience and frontoparietal (right and left). These networks correspond to RSNs described previously with high stability over time (Smith et al., 2009).

**Figure 2.**
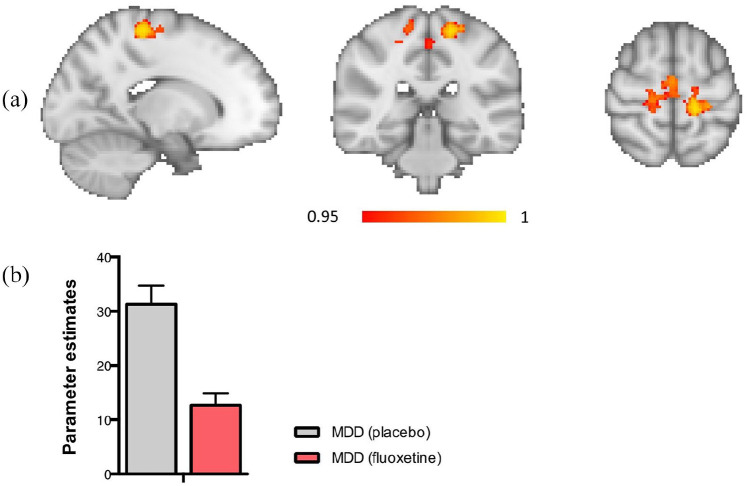
(a) Region of reduced connectivity with the motor network, the precentral/postcentral gyrus is shown. (b) Significantly reduced functional connectivity in the MDD group on fluoxetine compared with placebo between the motor network and a cluster containing the precentral and postcentral gyrus. *p* < 0.05.

Fluoxetine (vs. placebo) was found to reduce connectivity between the motor network and a cluster containing the precentral and postcentral gyrus (x = −16, y = −32, z = 62) (Figure 2). There were no significant group differences in connectivity in the DMN, or any of the other networks.

### Seed-based analysis

No significant group differences were found on the seed-based analysis, with either the left or the right amygdala.

## Conclusion

This is the first study, to our knowledge, to investigate the acute effects of fluoxetine on resting-state functional connectivity in depressed adolescents.

In contrast to previous studies that have demonstrated an effect of SSRIs on activity within the DMN with longer term treatment in both adults (Posner et al., 2013) and adolescents (Cullen et al., 2016), the current data suggest that a single dose of fluoxetine does not alter activity in this network in adolescent depression. This also stands in contrast to our previous study, in which we detected changes in fMRI response to angry facial expressions in the same group of adolescents (Capitão et al., 2019). This suggests that resting-state functional connectivity may be less affected early in treatment in young people. The reasons for this disparity are unclear but it is possible that the standard, single low dose used in the current study (10 mg) was not sufficient to induce alterations in the DMN and other depression-related networks, whilst the emotional processing task reported in our previous paper, with highly salient social stimuli, may have evoked stronger neural responses. Future studies should further explore this hypothesis, and also identify whether there may be early neural differences between patients who eventually respond to treatment (responders) vs. those who do not (nonresponders).

While we did not see the hypothesised change in DMN connectivity, we did see an unexpected group difference in the motor network. This is interesting in light of evidence showing that acute fluoxetine modulates cerebral motor activity (Loubinoux et al., 1999), however this finding should be clarified in future research.

Limitations of this study include the modest sample size. Our power calculation was based on previous task-based studies, hence it is possible that the sample size used here was insufficient to detect subtle drug effects on resting-state functional connectivity. Future studies with larger sample sizes are required.
